# Effectiveness of Positive Psychology Interventions for Cancer Survivors: A Systematic Review and Meta‐Analysis

**DOI:** 10.1002/cam4.71368

**Published:** 2026-01-07

**Authors:** Su Ann Yeoh, Alice Bowie, Tim Windsor, Hayley Russell, Saravana Kumar, Lisa Beatty

**Affiliations:** ^1^ Flinders University, College of Education, Psychology and Social Work, Flinders University Institute for Mental Health & Wellbeing Adelaide South Australia Australia; ^2^ Ovarian Cancer Australia Melbourne Victoria Australia; ^3^ Innovation, IMPlementation and Clinical Translation (IIMPACT in Health), Allied Health & Human Performance, University of South Australia Adelaide South Australia Australia

**Keywords:** cancer, interventions, oncology, positive psychology, positive psychology intervention, psychological interventions, psychological wellbeing, psycho‐oncology, psychosocial

## Abstract

**Objectives:**

This systematic review aimed to evaluate the effectiveness of positive psychology interventions (PPIs) that are theoretically grounded and tailored to individuals across various stages and types of cancer.

**Methods:**

Adhering to PRISMA guidelines, a comprehensive search of peer‐reviewed and gray literature was conducted across seven databases and supplementary sources, including Google and Google Scholar. Meta‐analyses were performed using standardized mean differences (Hedges' *g*) to assess the impact of PPIs on various psychological, physiological, and quality of life (QOL) outcomes. The methodological quality of included studies was appraised using the McMaster Critical Appraisal Tool, and the overall body of evidence was graded using the NHMRC FORM framework.

**Results:**

Eighteen studies involving 1382 participants were included. PPIs significantly improved psychological wellbeing domains such as post‐traumatic growth, positive emotions, engagement, meaning, and positive relationships, with effect sizes ranging from moderate to large (*p* < 0.05). Improvements were also observed in QOL and sexual functioning, though these outcomes were assessed in fewer studies. In contrast, limited or inconsistent effects were noted for accomplishment, psychological distress, physical functioning, and pain. High heterogeneity across studies highlighted variability in intervention designs and participant populations.

**Conclusions:**

PPIs hold promise as an integrative approach to psycho‐oncology care in enhancing psychological wellbeing among cancer survivors. While their effects on psychological distress and specific concerns like sexual dysfunction warrant further research, PPIs represent a valuable framework for supporting the multifaceted needs of cancer survivors. Standardization of interventions and integration into multidisciplinary care models are recommended to maximize their clinical utility.

## 1 Background

Globally, the prevalence of individuals living with cancer is projected to rise by 77% by 2050, reaching an estimated 35 million cases annually [1]. Despite advancements in detection and treatment, cancer diagnosis is associated with significant psychological challenges, including post‐traumatic stress symptoms, existential distress, depression, anxiety, and reduced quality of life (QOL) [2]. Additionally, cancer treatments often result in long‐term toxicities such as fatigue, physical appearance changes, and sexual dysfunction, contributing to substantial individual and health system burdens [2, 3, 4]. It is therefore essential to address and mitigate the negative sequelae while promoting wellbeing and quality of life among survivors.

A large body of evidence has demonstrated the effectiveness of psychosocial interventions tailored to cancer survivors to address the negative impacts [5, 6, 7]. Interventions vary from gold‐standard evidence‐based cognitive behavioral therapy and acceptance and commitment therapy, to body–mind practices of mindfulness meditation, breathlessness interventions, and music therapy [5, 6, 7]. However, they have predominantly focused on managing psychological distress and psychopathology symptoms of anxiety and depression, rather than distress prevention and promotion of psychological wellbeing. This trend is evident in a recent review by Semenenko et al. [6], encompassing 25 studies, where 64% of included studies prioritized the measurement of psychological distress or psychopathology outcomes. Similarly, a recent review of 13 studies on psychosocial interventions for ovarian cancer revealed that 92% of outcomes focused on psychological distress, while outcomes related to social wellbeing, psychological wellbeing and cancer‐specific concerns, such as fear of cancer recurrence and sexual dysfunction, were scarcely addressed [7]. This emphasis on distress‐based outcomes, while important, may result in the underrepresentation of other crucial aspects of a cancer survivor's overall mental wellbeing.

Traditionally, mental wellbeing has been operationalized based on the presence or absence of clinically relevant symptoms of psychopathology or psychological distress [8]. However, Keyes [9] proposed a dual‐continua model, conceptualizing mental wellbeing as comprising two interrelated dimensions: (1) the absence or presence of mental illness, ranging from severe psychological disorders to no mental health conditions; and (2) the level of psychological wellbeing, spanning from languishing (low wellbeing) to flourishing (high wellbeing). This model, recently validated by Stephens et al. [10], highlights that the absence of mental illness alone does not equate to positive wellbeing. Instead, mental wellbeing combines *both* the absence of distress with desirable psychological states, such as positive affect, purpose, and supportive relationships [10, 11]. Constructs such as psychological wellbeing, meaning in life, post‐traumatic growth, self‐compassion, and flourishing were identified as key determinants of successful cancer adjustment [12, 13, 14]. These constructs align with the principles of positive psychology [11], which aims to enhance human flourishing and supports the need for a more holistic approach that incorporates both the reduction of psychological distress and the promotion of wellbeing.

Positive psychology focuses on the science of human flourishing, promoting psychological, social, and emotional wellbeing [9, 11, 15]. This approach has been shown to positively influence health outcomes, including biological processes such as neuroendocrine and immune function, which may be directly related to disease progression and symptom management [16, 17]. One influential perspective on positive psychology is Seligman's (2008) [11] PERMA model, which identifies five key components of wellbeing: Positive Emotion, Engagement, Relationships, Meaning, and Accomplishment. Together, these elements foster flourishing rather than merely alleviating distress, offering a holistic approach to mental health. The model suggests that enhancing these aspects not only increases life satisfaction but can also support resilience, improve recovery trajectories, and foster treatment adherence in individuals facing health challenges, including cancer patients [11, 18, 19].

In recent years, several reviews have explored the effectiveness of positive psychology interventions (PPIs) for cancer survivors, with notable contributions by Casellas‐Grau et al. [12], Otto et al. [20], and Tian et al. [21]. Casellas‐Grau et al. [12] focused exclusively on breast cancer patients, while the other reviews included individuals with various cancer types. While these reviews highlight promising outcomes in enhancing wellbeing, resilience, and self‐efficacy, they also underscore key limitations. A lack of consistent definitions and theoretical grounding for PPIs complicates comparisons and generalizability across studies. For instance, interventions included in these reviews, such as mindfulness‐based stress reduction (MBSR), acceptance and commitment therapy (ACT), and spirituality‐based approaches, are rooted in philosophies and frameworks outside traditional positive psychology principles, such as PERMA [11]. This definitional variability limits clarity in distinguishing PPIs from other therapeutic modalities [22]. Methodological variability, including the exclusive reliance on randomized controlled trials (RCTs), further narrows the scope, potentially excluding valuable insights from alternative study designs and introducing publication bias by favoring published studies with positive findings [21]. Additionally, the focus on positive psychological outcomes, rather than also considering symptoms of distress, restricts the ability to assess interventions holistically [12, 21]. Moving forward, there is a need for broader, more rigorous reviews that incorporate diverse study designs, include both published and gray literature, and evaluate interventions for their dual capacity to reduce distress and promote wellbeing, thereby maximizing their overall impact on cancer care. Therefore, this review aims to address these gaps by critically assessing the effectiveness of PPIs, ensuring they are grounded in a clear theoretical framework and appropriately tailored to individuals across all stages and types of cancer.

## 2 Methods

The proposed systematic review was conducted and reported in accordance with the PRISMA‐SR guidelines [23]. The full protocol was developed prior and registered with the International Prospective Register of Systematic Review (PROSPERO): CRD42024546277.

### 2.1 Search Strategy

The search strategy aimed to identify published, peer‐reviewed studies, and gray literature. A preliminary search on MEDLINE and PsycINFO was performed to explore the present body of literature and establish key terms and medical subject headings (MeSH) within the field of interest. Search terms relating to *population* and *intervention* were developed (Table 1). To obtain independent verification, the search strategy was evaluated by, and feedback sought from an academic librarian at Flinders University.

**TABLE 1:** Key concepts and search terms.

Framework aspects	Search terms
Population	((cancer* or sarcoma* or leukemia* or tumour* or tumor* or carcinoma* or lymphoma* or myeloma* or melanoma* or adenocarcinoma* or glioma* or blastoma* or hepatoma* or mesothelioma* or teratoma*) adj2 (patient* or survivor*))
Intervention	(Positive* adj2 (psychology or psychotherap*))
Control/comparison	N/A
Outcome	N/A

Following the preliminary search, key terms and MESH were subsequently translated and applied across multiple databases: Ovid PsycINFO, Ovid MEDLINE, Ovid Embase, Ovid Emcare, CINAHL, Scopus, and the Cochrane Library. The searches were performed from inception to June 2024. Detailed search syntax for each database is provided in Appendix A (Tables A1, A2, A3, A4, A5, A6, A7). To mitigate the risk of publication bias, gray literature was also sought through targeted keyword searches on Google and Google Scholar, with the first 10 pages of search results reviewed [24]. A review of backward and forward citations for studies published from database inception until June 2024 was conducted. Organizational websites (such as Cancer Council Australia and websites of professional societies/associations in the United States, Europe, United Kingdom, and Australia) were searched for relevant publications. Search parameters were limited to English studies and human subjects, with no restrictions placed on publication date.

### 2.2 Eligibility Criteria

This review included studies involving cancer survivors or patients at any stage of the disease trajectory, including diagnosis, treatment, remission, and recurrence. Only studies that explicitly identified themselves as PPIs and were grounded in positive psychology theories or methodologies in the development of their interventions were eligible for inclusion. Studies incorporating any form of control group, such as waitlist controls, treatment as usual, or alternative psychological interventions, were included. No restrictions were placed on the type of outcome measures assessed. Detailed inclusion and exclusion criteria are presented in Table 2.

**TABLE 2:** Inclusion and exclusion criteria.

Concepts	Criteria	
	Inclusion	Exclusion
Population	Cancer patients/cancer survivors	Non‐cancer population Child/adolescence/pediatric cancer population Family members/carers/caregivers/dyads
Intervention	Positive psychology/psychotherapy intervention that adheres to positive psychology theoretical frameworks. Examples of positive psychology theoretical frameworks (e.g., Seligman's PERMA model, Keyes' dual‐continua model, Fredrickson's broaden‐and‐build theory, and Ryan and Deci's self‐determination theory)	Psychological/psychosocial interventions other than positive psychotherapy Positive psychology interventions that do not adhere or clearly delineate positive psychology theoretical frameworks (e.g., mindfulness, behavior activation). Physical and physiological interventions Dietary interventions Medicinal treatment Drug treatment Surgical treatment
Comparison	Waitlist or control group Treatment as usual Other forms of psychological intervention	N/A
Outcome	Psychological wellbeing Psychological Distress Mental illness Subjective wellbeing Clinical outcomes/Physical health Survival rates	N/A
Study	Human English Quantitative experimental studies	Animal Non‐English Qualitative studies Secondary evidence study Opinion articles Conference abstract

### 2.3 Study Selection Process

Studies were imported into EndNote 20 software to organize the retrieved results. Covidence software was subsequently used for the screening and selection of relevant articles after duplicate entries were removed. The screening process was conducted in two stages: an initial title and abstract screening, followed by full‐text screening based on the established eligibility criteria. Two independent reviewers (S.A.Y. and A.B.) conducted the screening, and any discrepancies or conflicts were resolved by consultation with a third independent reviewer (L.B.).

### 2.4 Assessment of Methodological Quality

All studies were independently appraised by two reviewers (S.A.Y. and S.K.) using a modified McMaster Quantitative Critical Appraisal Tool [25], with discrepancies resolved through discussion. This tool, widely used in systematic reviews [26, 27, 28], evaluates quantitative research quality across eight domains: study purpose, background literature, design, sample characteristics, outcome measures, intervention, results, and conclusions. Fourteen criteria were assessed, with responses categorized as ‘yes’ (1 point), ‘no,’ ‘not addressed’ (0 points), or ‘not applicable’ (excluded from the total score). Studies were not excluded based on quality but instead analyzed with consideration of potential bias and methodological rigor to inform the review's findings and interpretation.

### 2.5 Data Extraction

Data extraction was conducted using a standardized extraction form, detailed in Appendix B (Table B1). The extracted data included the following key components: reviewer initials, study number (as listed in Covidence), author, country of origin, study design, sample size and characteristics, intervention details, positive psychology theoretical framework, outcome measures, quantitative results, and study limitations. The extraction process was independently undertaken by two reviewers (S.A.Y. and A.B.), with each reviewer subsequently cross‐checking the other's work to ensure accuracy and consistency. Discrepancies in data extraction between reviewers were resolved by another reviewer (L.B.).

### 2.6 Data Synthesis

To synthesize data, results were categorized into three distinct domains. The first domain encompassed *positive psychology outcomes*, including post‐traumatic growth, a key aspect of flourishing, and the five elements of Seligman's Positive Psychology Framework (PERMA), specifically: positive emotions, meaning, engagement, positive relationships, and accomplishment. Additionally, this domain included measures of subjective wellbeing, such as quality of life (QOL). These constructs were collectively analyzed to determine their contribution to psychological wellbeing and growth, with effect sizes calculated to assess their impact. The second domain focused on *psychological disorders and distress*, such as depression, psychological distress, and post‐traumatic stress, synthesizing studies that examined interventions aimed at reducing these negative mental health outcomes. The third domain involved *physiological outcomes*, such as physical and sexual functioning, and pain, examining the broader health effects of interventions and the link between psychological wellbeing and physiological health. Outcomes measured by fewer than three studies were narratively synthesized within their respective domains.

Meta‐analysis was performed using the Comprehensive Meta‐Analysis Version 4 software. Standardized mean differences (SMD) with 95% confidence intervals (CI) were exclusively used in this review, as most studies employed different measurement scales to assess the same outcomes. Given most studies included small sample sizes, Hedges' *g* was chosen as the appropriate effect size measure due to its correction for small sample bias. The within‐group mean change (Mean) was calculated using the formula “Mean change = Mean after − Mean baseline,” and the standard deviation (SD) of the mean change was determined using the formula “SD change = √[SD^2^ baseline + SD^2^ after − (2 * correlation * SD baseline * SD after)].” Hedges' *g* values were interpreted as 0.8 for large effects, 0.5 for moderate effects, and 0.2 for small effects [29]. Heterogeneity between studies was assessed using the *I* ^2^ statistic. For *I* ^2^ ≤ 50%, heterogeneity was considered low, and a fixed‐effects model was applied. If *I* ^2^ exceeded 50%, indicating high heterogeneity, a random‐effects model was used. For reporting purposes, *I* ^2^ values were categorized as follows, based on the guidelines provided by Higgins et al. [30]: 0%–24% as no heterogeneity, 25%–50% as low heterogeneity, 51%–75% as moderate heterogeneity, and 76%–100% as high heterogeneity. Sensitivity analyses were conducted by removing each study one at a time to see if it caused significant changes in the pooled effect size. Based on these thresholds, a random‐effects model was planned a priori and applied where heterogeneity was moderate to high, to account for between‐study variability and enhance generalisability.

Additionally, the overall body of evidence was graded using four of the five National Health and Medical Research Council (NHMRC) FORM framework [31] components: (1) Evidence base, (2) Consistency, (3) Clinical impact, and (4) Generalisability. Component 5 (Applicability to the Australian healthcare context) was not evaluated, given this review is intended for international health care [32]. The data synthesis process was conducted by one reviewer (S.A.Y.) with consultation between reviewers (L.B./T.W./S.K.).

## 3 Results

**FIGURE 1:**
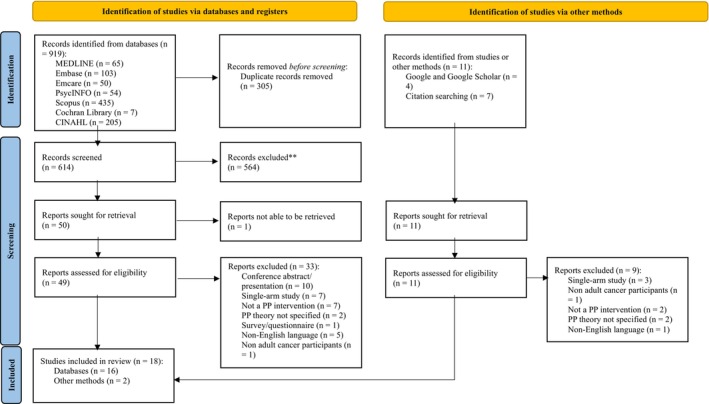
PRISMA flow chart.

The literature search yielded a total of 930 records, including 919 identified from electronic databases and 11 from gray literature and citation searches (see Figure 1). After removing duplicates, 614 records underwent title and abstract screening with 564 records excluded for not meeting the predefined inclusion criteria. Of the 50 full‐text reports sought for retrieval, one could not be obtained and was excluded from the screening process. The remaining 49 were assessed for eligibility, resulting in 33 exclusions (see Figure 1 for reasons). Ultimately 18 studies were included in this review.

### 3.1 Characteristics of Included Studies

The 18 included studies were published between 2009 and 2023 with the majority (61%) published from 2020 onwards (Table 3). The studies were diverse in design, population, and intervention characteristics, and were conducted across seven countries, including Iran (*n* = 5) [33, 34, 35, 36, 37], China (*n* = 4) [38, 39, 40, 41], Iraq (*n* = 3) [42, 43, 44], Spain (*n* = 3) [45, 46, 47], the United States (*n* = 1) [48], the United Kingdom (*n* = 1) [49], and Portugal (*n* = 1) [50]. Half the included studies employed quasi‐experimental designs [34, 35, 36, 40, 41, 42, 43, 44, 46], often utilizing pre‐test and post‐test methodologies, while the other half implemented randomized controlled trials (RCTs) [33, 37, 38, 39, 41, 45, 47, 48, 49]. Sample sizes ranged from small (*n* = 30) [34, 36], to larger‐scale trials (*n* = 175) [45], with a total of 1382 participants across all studies. The majority of participants were female, with several studies (*n* = 6) [33, 35, 38, 41, 42, 45] focusing exclusively on patients with breast cancer.

**TABLE 3:** Study characteristics.

Study characteristics		Sample characteristics		Intervention characteristics			Measures	
Author, year, & country	Design	Sample size	Characteristics	Positive psychology theory/framework	Mode, length × frequency (follow‐up)	Intervention	Outcome measures	Outcome domain
Alrazaq et al. [43] 2022 Iraq	Quasi‐experimental (pre‐test, post‐test)	*n* = 75 (CG = 25, PPI = 25, GI = 25)	Age: Under 40 (*n* = 17, 22.6%), 41–50 (*n* = 27, 36.0%), 50–65 (*n* = 31, 41.3%) Marital status: Single (*n* = 16, 21.3%), married (*n* = 59, 78.7%) Gender: Male (*n* = 36, 48.0%), Female (*n* = 39, 52.0%) Cancer Type: Lung cancer (*n* = 75, 100%)	Positive psychology wellbeing theory	Face to face, 90 min × 8 sessions (no follow‐up)	Group positive‐oriented psychotherapy	Psychological Wellbeing Scale	Autonomy ↑, environmental mastery ↑, personal growth ↑, positive relationships with others ↑, life purpose ↑, and self‐acceptance ↑
Al‐Zubaidi et al. [42] 2022 Iraq	Quasi‐experimental (pre‐test, post‐test, follow‐up)	*n* = 100 (CG = 50, IG = 50)	Age: 35–40 (*n* = 17, 17.0%), 41–50 (*n* = 43, 43.0%), 51–55 (*n* = 40, 40.0%) Marital status: Single (*n* = 16, 16.0%), married (*n* = 84, 84.0%) Gender: Female (*n* = 100, 100%) Cancer type: Breast cancer (*n* = 100, 100%)	Integrative positive psychology theory (with hope theory)	Face to face, 90 min × 8 sessions (2‐months follow‐up)	Group positive psychology intervention	Snyder Hope Scale, the Self‐Compassion Scale, and the Post‐Traumatic Growth Inventory (PTGI)	Hope ↑, self‐compassion ↑, post traumatic growth ↑, PTGI ↑
Berg et al. [48] 2019 United States	Pilot RCT	*n* = 56 (CG = 18, IG = 38)	Age: mean = 32.6 years Marital status: Married/living with partner (*n* = 38, 67.9%), other (*n* = 18, 32.1%) Gender: Male (*n* = 14, 25.0%), Female (*n* = 42, 75.0%) Cancer type: Breast (*n* = 16, 28.6%), melanoma (*n* = 9, 16.1%), leukemia/lymphoma (*n* = 7, 12.5%), sarcoma (*n* = 5, 8.9%), colorectal (*n* = 3, 5.4%), testicular (*n* = 3, 5.4%), cervical (*n* = 2, 3.6%)	Snyder (2009) conceptualisation of hope theory	Telehealth & self‐administered telephone app, NR × 8 sessions (6‐months follow‐up)	Individual hope‐based intervention, (Achieving Wellness After Kancer in Early life (AWAKE))	Adult Trait Hope Scale, RAND Medical Outcome Study 36‐item short form health survey (SF‐36), Functional Assessment of Cancer therapy‐general (FACT‐G), and Patient Health Questionnaire—9 item (PHQ‐9)	Hope ↑, health behaviors ↑, depression ↓
Cerezo et al. [45] 2014 Spain	RCT	*n* = 175 (CG = 88, IG = 88)	Age: mean = CG (49.4 years), IG (50.7 years) Marital status: Single (*n* = 38, 21.7%), married (*n* = 105, 60.0%), separated/divorced (*n* = 27, 15.4%), widowed (*n* = 5, 2.9%) Gender: Female (*n* = 175, 100%) Cancer type: Breast cancer (*n* = 175, 100%)	Seligman positive psychology theory	Face to face, 120 min × 14 sessions (1‐month follow‐up)	Group positive psychology intervention	Satisfaction with Life Scale, Affectivity Scale, Trait Meta‐Mood Scale‐24 (TMMS‐24), Life Orientation Test Revised (LOT‐R), Connor‐Davidson Resilience Scale (CD‐RISC), Rosenberg Self‐Esteem Scale	Cognitive wellbeing ↑, total affect ↑, positive affect ↑, emotional intelligence ↑, optimism ↑, resilience, self‐esteem ↑, happiness ↑
Dowlatabadi et al. [33] 2016 Iran	RCT	*n* = 33 (CG = 17, IG = 16)	Age: mean = 36.6 years Marital status: NR Gender: Female (*n* = 33, 100%) Cancer type: Breast cancer (*n* = 33, 100%)	Seligman positive psychology theory	Face to face, 90 min × 10 sessions (No follow‐up)	Group positive psychotherapy	Beck Depression Inventory (BDI‐II), Oxford Happiness Inventory (OHI)	Depression ↓, happiness ↑
Fadaei‐Tirani et al. [34] 2023 Iran	Quasi‐experimental	*n* = 30 (CG = 15, IG = 15)	Age: mean = Male (39.2 years), Female (33.7 years) Marital status: NR Gender: NR Cancer type: Leukemia (*n* = 30, 100%)	Integrative positive psychology theory (with learned optimism)	Face to face, 90 min × 14 sessions (1‐month follow‐up)	Group positive psychology training	Distress Tolerance Scale (DTS), Life Orientation Test (LOT)	Emotional distress tolerance ↑, optimism ↑
Fang et al. [38] 2023 China	RCT	*n* = 82 (CG = 41, IG = 41)	Age: ≤ 45 years (*n* = 38, 46.3%), > 45 years (*n* = 44, 53.7%) Marital status: Married (*n* = 74, 90.2%), divorced (*n* = 8, 9.8%) Gender: Female (*n* = 82, 100%) Cancer type: Breast cancer (*n* = 82, 100%)	PERMA framework	Face to face, 40–50 min × 7 sessions (No follow‐up)	Group PERMA model based positive psychology intervention program	Self‐rating anxiety scale (SAS), Self‐rating depression scale (SDS), Functional assessment of cancer therapy‐breast (FACT‐B)	Anxiety ↓, depression ↓, QOL ↑, physiological functioning ↑, social/family condition ↑, emotional condition ↑, functional condition ↑, FACT‐B ↑
Haseli et al. [35] 2023 Iran	Quasi‐experimental	*n* = 45 (CG = 15, CFT = 15 IG = 15)	Age: mean = CG (50.1 years), CFT (48.3 years), IG (49.5 years) Marital status: NR Gender: Female (*n* = 45, 100%) Cancer type: Breast cancer (*n* = 45, 100%)	Integrative positive psychology theory (with psychological wellbeing and self‐worth)	Face to face, 60 min × 8 sessions (No follow‐up)	Group positive psychotherapy	Self‐worth Questionnaire, Psychological Wellbeing Questionnaire	Self‐acceptance ↑, psychological wellbeing ↑, positive relationships ↑, purpose in life ↑, personal growth ≈, self‐worth ↑
Kadhim et al. [44] 2022 Iraq	Quasi‐experimental	*n* = 50 (CG = 25, IG = 25)	Age: mean = 66.8 years Marital status: NR Gender: Male (*n* = 50, 100%) Cancer type: Prostate cancer (*n* = 50, 100%)	Seligman positive psychology theory	Face to face, 120 min × 8 sessions (No follow‐up)	Group positive psychotherapy	Differentiation of Self Inventory‐Revised (DSI‐R)	Social relationships ↑, emotional reactivity ↑, self‐differentiation ↑
Louro et al. [50] 2016 Portugal	Pilot quasi experimental	*n* = 44 (CG = 24, IG = 20)	Age: < 50 years (*n* = 9, 20.5%), 50–59 years (*n* = 14, 31.8%), ≥ 60 years (*n* = 21, 47.7%) Marital status: Single (*n* = 3, 6.8%), married (*n* = 33, 75.0%), divorced/separated (*n* = 5, 11.3%), widowed (*n* = 3, 6.8%) Gender: Male (*n* = 29, 65.9%), Female (*n* = 15, 34.1%) Cancer type: Colon cancer (*n* = 30, 68.2%), rectal (*n* = 14, 31.8%)	Integrative positive psychology theory (with cognitive behavioral orientation)	Face to face, NR × 4 sessions (1‐month follow‐up)	Individual Enhancing Positive Psychology Procedure (EPEP)	Positive and Negative Affect Scale (PANAS; portugese version), Cancer Quality of Life Questionnaire Core 30 (EORTC QLQ‐C30, version 3), Psychological Treatment Evaluation	Positive affect ↑, negative affect ≈, global health status ↑, physical functioning ↑, social functioning ↑, emotional functioning ≈, cognitive functioning ≈
Meibodi et al. [36] 2021 Iran	Quasi‐experimental	*n* = 30 (CG = 15, IG = 15)	Age: mean = CG (41.5 years), IG (46.4 years) Marital status: NR Gender: Male (*n* = 12, 40.0%), Female (*n* = 18, 60.0%) Cancer type: NR	Seligman positive psychology theory	Face to face, 120 min × 8 sessions (No follow‐up)	Individual positive psychotherapy	Oxford Happiness‐Depression Questionnaire	Character strength ↑, pleasure ↑, commitment ↑, happiness ↑
Ochoa‐Arnedo et al. [47] 2021 Spain	RCT	*n* = 140 (CG = 73, IG = 67)	Age: mean = CG (49.7 years), IG (50.8 years) Marital status: Single (*n* = 12, 8.6%), married (*n* = 106, 75.7%), separated/divorced (*n* = 17, 12.1%), widowed (*n* = 5, 3.6%) Gender: NR Cancer type: Breast (*n* = 117, 83.6%), colorectal (*n* = 7, 5.0%), gynecological (*n* = 5, 3.6%), others (*n* = 11,7.9%)	Integrative positive psychology theory (with humanistic existential perspective)	Face to face, 90 min × 12 sessions (12‐month follow‐up)	Group positive psychotherapy	The Post‐traumatic Stress Disorder Checklist‐Civilian version (PCL‐C), Hospital Anxiety and Depression Scale (HADS), The Post‐traumatic Growth Inventory (PTGI)	Post‐traumatic stress ↓, distress ↓, post‐traumatic growth (PTGI) ↑, HADS ↓
Ochoa et al. [46] 2017 Spain	Quasi‐experimental	*n* = 126 (CG = 53, IG = 73)	Age: mean = CG (48.5 years), IG (48.9 years) Marital status: Single (*n* = 9, 7.1%), married (*n* = 101, 82.1%), separated/divorced (*n* = 13, 10.3%), widowed (*n* = 3, 2.4%) Gender: Female (*n* = 126, 100%) Cancer type: Breast (*n* = 112, 88.9%), uterine corpus (*n* = 3, 2.4%), lymphoma (*n* = 4, 3.2%), colon (*n* = 2, 1.6%), leukemia (*n* = 2, 1.6%), ovarian (*n* = 2, 1.6%), rectum (*n* = 1, 0.8%)	Integrative positive psychology theory (with orgasmic valuing theory)	Face to face, 90–120 min × 12 sessions (12‐month follow‐up)	Group positive psychotherapy	The Hospital Anxiety and Depression Scale (HADS), The Posttraumatic Stress Disorder Checklist‐Civilian version (PCL‐C), The Posttraumatic Growth Inventory (PTGI), The Extreme Life Events Inventory	Negative mood ↓, stress ↓, post‐traumatic stress ↓, post‐traumatic growth ↑, PCL‐C ↓, HADS ↓, PTGI ↑
Ramachandra et al. [49] 2009 United Kongdom	RCT	*n* = 46 (CG = 23, IG = 23)	Age: mean = 66.9 years Marital status: NR Gender: Male (*n* = 24, 52.2%), female (*n* = 22, 47.8%%) Cancer type: Breast cancer (*n* = 22, 47.8%), prostate cancer (*n* = 24, 52.2%)	Integrative positive psychology theory (with mindfulness and wellbeing)	Self‐administered, NR × NR (No follow‐up)	Individual wellbeing intervention	WHO Quality of Life Scale BREF (WHOQOL–BREF), Hospital Anxiety and Depression Scale (HADS), The Social and Occupational Functioning Assessment Scale (SOFAS), Life Orientation Test (Revised) (LOT‐R), Ten‐item Personality Inventory (TIPI)	Quality of life (WHOQOL–BREF) ↑, anxiety and depression (HADS) ↓, overall functioning (SOFAS) ≈
Saeedi et al. [37] 2019 Iran	RCT	*n* = 61 (CG = 31, IG = 30)	Age: mean = CG (47.9 years), IG (47.5 years) Marital status: Single (*n* = 5, 8.2%), married (*n* = 48, 78.7%), widowed/divorced (*n* = 8, 13.1%) Gender: Male (*n* = 4, 6.6%), female (*n* = 57, 93.4%) Cancer type: Breast (*n* = 37, 60.7%), others (*n* = 24, 39.3%)	Seligman positive psychology theory	Face to face, 90 min × 8 sessions (No follow‐up)	Group positive psychotherapy	Life Attitude Profile‐Gary Reker Questionnaire	Life purpose ↑, meaning in life ↑
Shi et al. [39] 2020 China	RCT	*n* = 91 (CG = 45, IG = 46)	Age: 18–30 years (*n* = 3, 3.3%), 31–40 years (*n* = 31, 34.1%), 41–50 years (*n* = 57, 62.6%) Marital status: Single (*n* = 4, 4.4%), married (*n* = 46, 50.5%), divorced (*n* = 9, 9.9%), widowed (*n* = 2, 2.2%) Gender: Female (*n* = 91, 100%) Cancer type: Cervical cancer (*n* = 91, 100%)	PERMA framework	Self‐administered, 45–60 min × 8 session (No follow‐up)	Individual positive psychology intervention	The Female Sexual Function Index, The Self‐rating Depression Scale, The Index of Well‐Being	Depression ↑, subjective wellbeing ↑, sexual functioning ↑
Tu et al. [40] 2021 China	Quasi‐experimental	*n* = 100 (CG = 50, IG = 50)	Age: mean = CG (57.3 years), IG (56.4 years) Marital status: Married (*n* = 86, 86.0%), divorced/widowed (*n* = 14, 14.0%) Gender: Male (*n* = 56, 56.0%), female (*n* = 44, 44.0%) Cancer type: Lung cancer (*n* = 100, 100%)	PERMA framework	Face to face, 30–40 min × NR (No follow‐up)	Individual positive psychology intervention + exercise	Herth hope index (HII), Posttraumatic growth inventory (PTGI), Self‐rating anxiety scale (SAS), Cancer fatigue scale (CFS)	Optimism ↑, autonomy ↑, positive relationships ↑, hope ↑, post‐traumatic growth ↑, anxiety ↓, depression ↓, fatigue ↓, PTGI ↑
Zhang et al. [41] 2023 China	RCT	*n* = 98 (CG = 49, IG = 49)	Age: mean = CG (48.7 years), IG (47.4 years) Marital status: Single (*n* = 6, 6.1%), married (*n* = 92, 93.9%) Gender: Female (*n* = 98, 100%) Cancer type: Breast cancer (*n* = 98, 100%)	Integrative positive psychology theory (cognitive adaptation theory)	Self‐administered, 30 min × 4 sessions (1‐month follow‐up)	Individual, family‐centred positive psychology intervention	Connor Davidson Resilience Scale (CD‐RISC), The Herth Hope Index (HHI), Perceived Benefits of Diagnosis and Treatment of Breast Cancer (PB‐DT‐BC), Functional Assessment of Cancer Therapy‐Breast (FACT‐B)	Resilience ↑, hope ↑, overall functioning ↑, optimism ↑, positive attitude ↑, autonomy ↑, social relationship ↑, emotional state ↑, FACT‐B ↑

*Note:* ↑ = significant increase compared to control, ↓ = significant decrease compared to control, ↑ = non‐significant increase compared to control, ↓ = non‐significant decrease, ≈ = no difference between intervention and control.

Abbreviations: CFT = compassion focused therapy, CG = control group, GI = gestalt intervention, IG = intervention group, NR = not reported, QOL = quality of life.

#### 3.1.1 Intervention Characteristics

Interventions ranged between four and 14 sessions, entailing psychoeducation (on acceptance of the disease and challenging negative thoughts), cultivating positive relationships, completing gratitude worksheets, engaging with pleasurable activities, recognition of personal strength, personal growth from the illness experience, and goal setting and attainment [33, 34, 36, 37, 38, 40, 42, 43, 44, 45, 46, 47, 48, 50] (Table 3). The intervention modality varied across studies but typically involved face‐to‐face group sessions [33, 34, 35, 36, 37, 38, 40, 42, 43, 44, 45, 46, 47, 50], with session lengths ranging from 30 [40, 41] to 120 min [36, 44, 45, 46]. Follow‐up periods ranged from providing no follow‐up (56%) [33, 35, 36, 37, 38, 39, 40, 43, 44, 49], 1 month follow‐up [34, 41, 45, 50], to 12 months post‐intervention [46, 47]. Common theoretical frameworks guiding the interventions included Seligman's Positive Psychology therapeutic approach [33, 36, 37, 44, 45], the PERMA framework [38, 39, 40], and Snyder's Hope Theory [48].

#### 3.1.2 Outcome Measures

The included studies employed diverse measures to assess positive psychology components, psychological distress, and physiological outcomes (Table 3). Within positive psychology, post‐traumatic growth was assessed using tools such as the Post‐Traumatic Growth Inventory (PTGI) and Connor‐Davidson Resilience Scale (CD‐RISC) [35, 40, 41, 42, 43, 45, 46, 47]. Positive emotions and engagement were measured using instruments like the Positive and Negative Affect Scale (PANAS), RAND SF‐36, and Cancer Quality of Life Questionnaire (EORTC QLQ‐C30) [36, 38, 45, 48, 50]. Positive relationships, meaning, and accomplishment were evaluated with scales such as the FACT‐B, Differentiation of Self Inventory‐Revised (DSI‐R), and Adult Trait Hope Scale, and [35, 38, 40, 41, 43, 44]. Measures of QOL and psychological wellbeing included the FACT‐B, WHOQOL‐BREF, and Psychological Wellbeing Questionnaire [35, 38, 49]. For psychological distress, depression and anxiety were assessed using the Beck Depression Inventory (BDI‐II), Self‐Rating Depression Scale (SDS), Patient Health Questionnaire‐9 (PHQ‐9) and Hospital Anxiety and Depression Scale (HADS) [33, 34, 38, 40, 46, 47, 48]. Post‐traumatic stress was measured in two studies using the Posttraumatic Stress Disorder Checklist‐Civilian version (PCL‐C) [46, 47]. Within physiological outcomes, physical functioning, sexual functioning, and pain were measured using the RAND SF‐36, FACT‐B, EORTC QLQ‐C30, and Female Sexual Function Index (FSFI) [38, 39, 41, 48, 50].

### 3.2 Methodological Quality

Overall, the quality of the studies ranged from moderate to high, with 15 studies classified as high quality [33, 34, 36, 37, 38, 39, 40, 41, 42, 43, 45, 46, 47, 48, 50] and three as moderate quality [35, 44, 49]. All studies consistently met the following core criteria: clearly stating their purpose (criterion 1), justifying the importance and providing sufficient background literature to frame the study rationale (criterion 2), reporting results in terms of statistical significance (criterion 7a), and providing appropriate conclusions relative to the results obtained (criterion 8) (Table 4). All but one study [49] adequately addressed reliability and validity of outcome measure tools (Criterion 5a and 5b); therefore, the majority of studies present with reduced risk of both random and systematic bias.

**TABLE 4:** McMaster critical appraisal assessment.

Study	McMaster critical appraisal criterion															
	1	2	4b	4c	5a	5b	6a	6b	6c	7a	7b	7c	7d	8	Score	%
Alrazaq et al. 2022	Y	Y	Y	N	Y	Y	Y	NAD	N	Y	Y	Y	N	Y	10	71
Al‐Zubaidi et al., 2022	Y	Y	Y	Y	Y	Y	Y	NAD	N	Y	Y	Y	N	Y	11	79
Berg et al. 2019	Y	Y	Y	Y	Y	Y	Y	NAD	NAD	Y	Y	N	Y	Y	11	79
Cerezo & Ortiz‐Tallo, 2014	Y	Y	Y	Y	Y	Y	Y	Y	NAD	Y	Y	Y	Y	Y	13	93
Dowlatabadi et al. 2016	Y	Y	Y	N	Y	Y	Y	N	NAD	Y	Y	N	Y	Y	10	71
Fadaei‐Tirani et al., 2023	Y	Y	N	N	Y	Y	Y	N	Y	Y	Y	Y	N	Y	10	71
Fang et al., 2023	Y	Y	Y	N	Y	Y	Y	Y	Y	Y	Y	N	N	Y	11	79
Haseli et al., 2022	Y	Y	N	N	Y	Y	N	N	Y	Y	N	Y	N	Y	8	57
Kadhim et al., 2022	Y	Y	N	N	Y	Y	Y	N	N	Y	Y	Y	N	Y	9	64
Louro et al. 2016	Y	Y	Y	N	Y	Y	Y	Y	N	Y	Y	N	Y	Y	11	79
Meibodi et al., 2021	Y	Y	N	N	Y	Y	Y	N	Y	Y	Y	Y	N	Y	10	71
Ochoa‐Arnedo et al., 2021	Y	Y	Y	N	Y	Y	Y	Y	Y	Y	Y	Y	Y	Y	13	93
Ochoa et al., 2017	Y	Y	Y	N	Y	Y	Y	Y	N	Y	Y	Y	Y	Y	12	86
Ramachandra et al., 2009	Y	Y	N	N	N	N	Y	N	Y	Y	Y	N	Y	Y	8	57
Saeedi et al., 2019	Y	Y	Y	N	Y	Y	Y	N	Y	Y	Y	N	Y	Y	11	79
Shi et al., 2020	Y	Y	Y	Y	Y	Y	Y	Y	Y	Y	Y	Y	Y	Y	14	100
Tu et al., 2021	Y	Y	Y	N	Y	Y	Y	N	Y	Y	Y	N	N	Y	10	71
Zhang et al., 2023	Y	Y	Y	Y	Y	Y	Y	Y	Y	Y	Y	N	Y	Y	13	93

Abbreviations: N = no, NAD = not addressed, Y = yes.

However, the majority of studies failed to justify the sample size utilised [33, 34, 35, 36, 37, 38, 40, 44, 46, 47, 49, 50] and provide sufficient description of the participants within the study [34, 35, 36, 44, 49] (Criterion 4b and 4c) as illustrated in Table 4. Contamination (criterion 6b) was also frequently unaddressed [42, 43, 48] or control measures were not implemented [33, 34, 35, 36, 37, 40, 44, 49], which may reduce internal validity and limit the ability to draw accurate conclusions about intervention effects. Nearly half the studies did not report dropout rates (criterion 7d), introducing potential attrition bias [34, 35, 36, 38, 40, 42, 43, 44].

In summary, while the majority of studies demonstrated methodological soundness in terms of purpose, literature review, and statistical reporting, there were frequent gaps in areas such as sample size justification, participant descriptions, and the control of contamination, all of which may affect the interpretation and reliability of their findings. The inclusion of both high‐ and moderate‐quality studies in this review enabled a comprehensive synthesis but necessitates caution in interpreting the results.

### 3.3 Effectiveness of Positive Psychology Interventions (PPIs)

#### 3.3.1 Positive Psychology Outcomes

##### 3.3.1.1 Post Traumatic Growth

**FIGURE 2:**
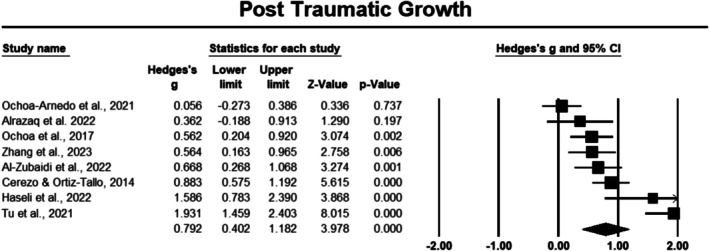
Forest plot: Effect of PPIs on post traumatic growth. Study weights are reflected in the size of the squares.

Eight studies evaluated the effect of PPIs on post‐traumatic growth in 859 cancer participants [35, 40, 41, 42, 43, 45, 46, 47]. The meta‐analysis revealed a significant positive effect of PPIs on post‐traumatic growth, with an overall effect size (Hedges' *g*) of 0.729 and a 95% confidence interval (CI) of 0.402 to 1.182 (Figure 2). This indicates a moderate to large effect, and the result was statistically significant (*p* < 0.05). Sensitivity analysis reflected stable results, revealing no change in effect size when each study was removed from the analysis. However, the heterogeneity was high (*I* ^2^ = 85.782%), suggesting substantial variability among the included studies.

##### 3.3.1.2 Components of PERMA Model

###### 3.3.1.2.1 Positive Emotions

**FIGURE 3:**
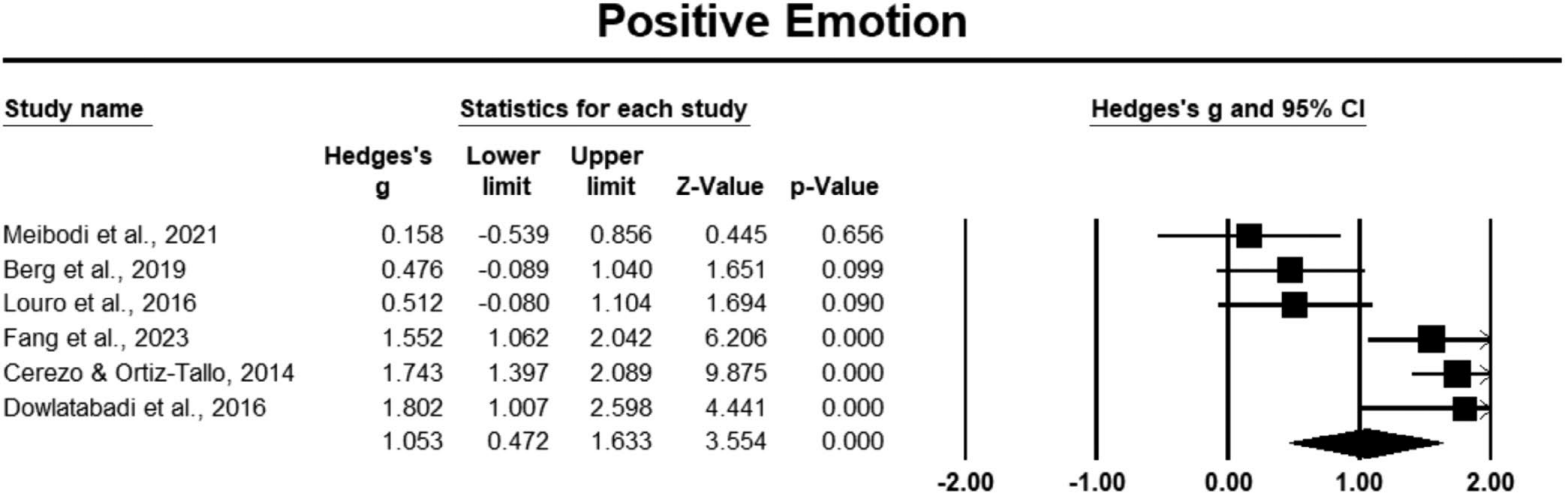
Forest plot: Effect of PPIs on positive emotions. Study weights are reflected in the size of the squares.

Six studies consisting of 420 cancer survivors observed significant positive effects [33, 36, 38, 45, 48, 50]. Positive emotions had a large effect size of 1.053 (Hedges' *g*), with a 95% CI of 0.427 to 1.633, and this result was significant (*p* < 0.05) (Figure 3). The sensitivity analysis demonstrated consistent results, indicating that the effect size remained stable when each study was sequentially excluded from the analysis. However, heterogeneity was notably high, with an *I* ^2^ value of 85.148%, indicating substantial variability across the studies.

###### 3.3.1.2.2 Engagement

**FIGURE 4:**
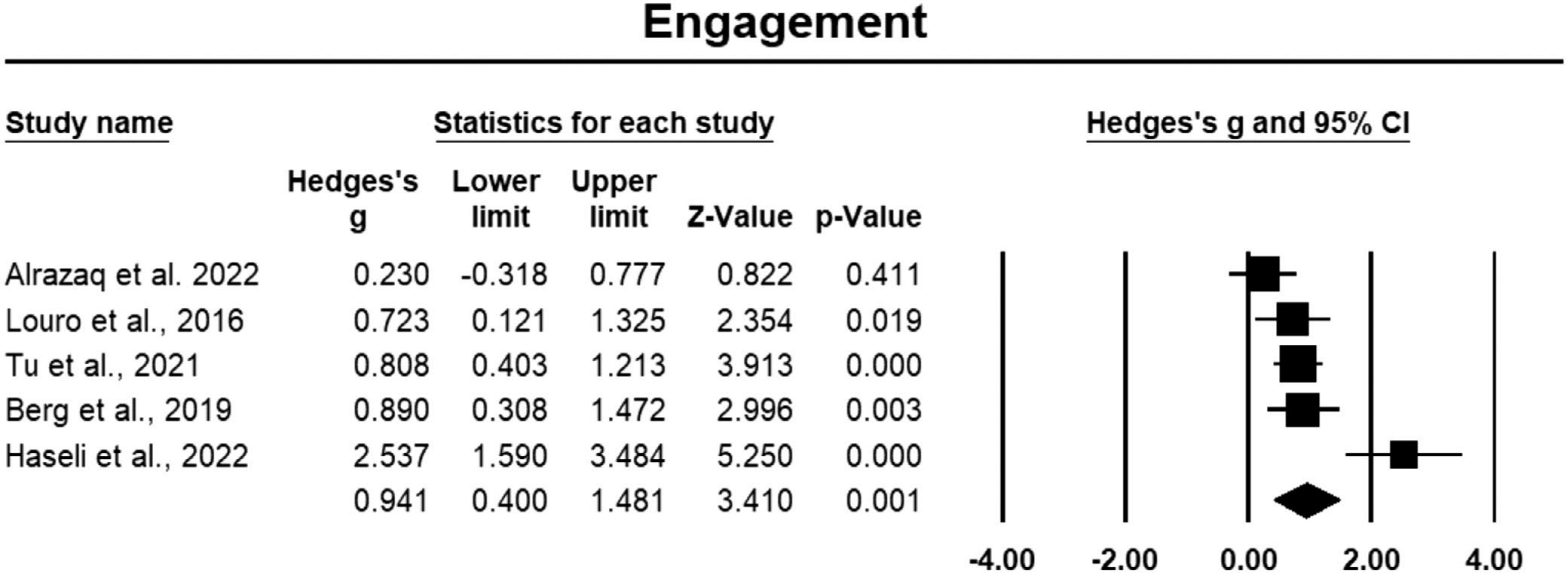
Forest plot: Effect PPIs on engagement. Study weights are reflected in the size of the squares.

The engagement component was assessed in five studies involving a total of 354 cancer participants. Engagement demonstrated a large effect size of 0.941 (Hedges' *g*), with a 95% CI of 0.400 to 1.481, which was statistically significant (*p* < 0.05) (Figure 4). The sensitivity analysis showed stable outcomes, with no variation in effect size observed when individual studies were sequentially removed from the analysis. However, heterogeneity was considerable, with an *I* ^2^ value of 76.796%, indicating substantial variability across the studies.

###### 3.3.1.2.3 Positive Relationships

**FIGURE 5:**
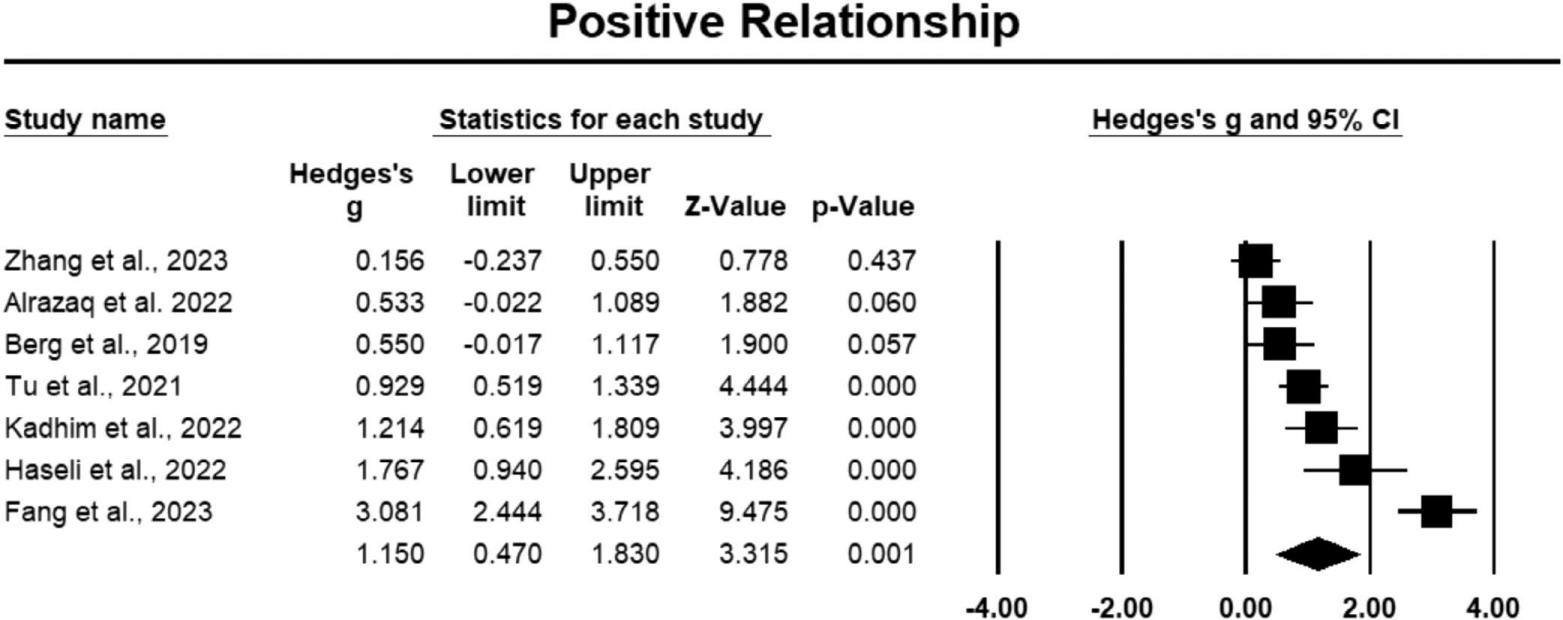
Forest plot: Effect PPIs on positive relationship. Study weights are reflected in the size of the squares.

The positive relationships component was assessed in seven studies involving 464 cancer participants [35, 38, 40, 41, 43, 44, 48]. Positive relationships demonstrated the largest effect size among all positive psychology components, with a Hedges' *g* of 1.150 and a 95% CI ranging from 0.470 to 1.830, which was statistically significant (*p* < 0.05) (Figure 5). Sensitivity analysis confirmed stable results, with the effect size unaffected by the exclusion of any single study. However, the heterogeneity was high, with an *I* ^2^ value of 91.059%, reflecting substantial variability across the studies.

###### 3.3.1.2.4 Meaning and Accomplishment

**FIGURE 6:**
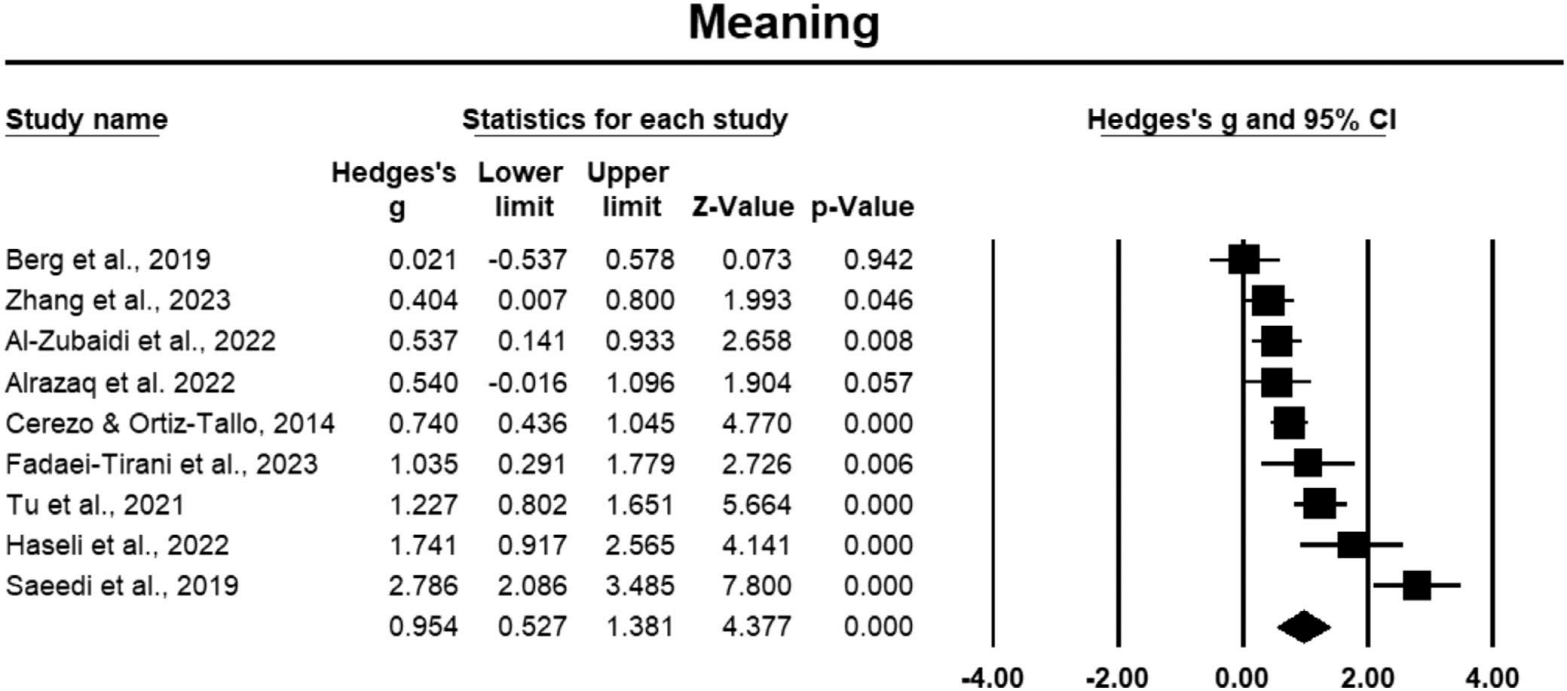
Forest plot: Effect PPIs on meaning. Study weights are reflected in the size of the squares.

Meaning was measured in nine studies involving a total of 440 cancer participants [12, 34, 35, 37, 40, 41, 42, 43, 48]. The analysis revealed a large effect size of 0.954 (Hedges' *g*), with a 95% CI of 0.527 to 1.381, which was statistically significant (*p* < 0.05) (Figure 6). Stability in the effect size was confirmed through sensitivity analysis, as removing individual studies did not impact the results. However, heterogeneity was high, with an *I* ^2^ value of 85.242%, indicating considerable variability across the studies.

Accomplishment was only examined in a single study focused on health behavior goals involving 56 cancer survivors [48], and did not demonstrate significant improvements following the positive psychology intervention.

###### 3.3.1.2.5 Subjective Wellbeing: Quality of Life

Quality of life was evaluated in two studies involving 128 cancer participants [38, 49], and demonstrated significant positive improvement in quality of life in both studies following a PPI.

#### 3.3.2 Psychological Disorders and Distress Outcomes

##### 3.3.2.1 Depression

**FIGURE 7:**
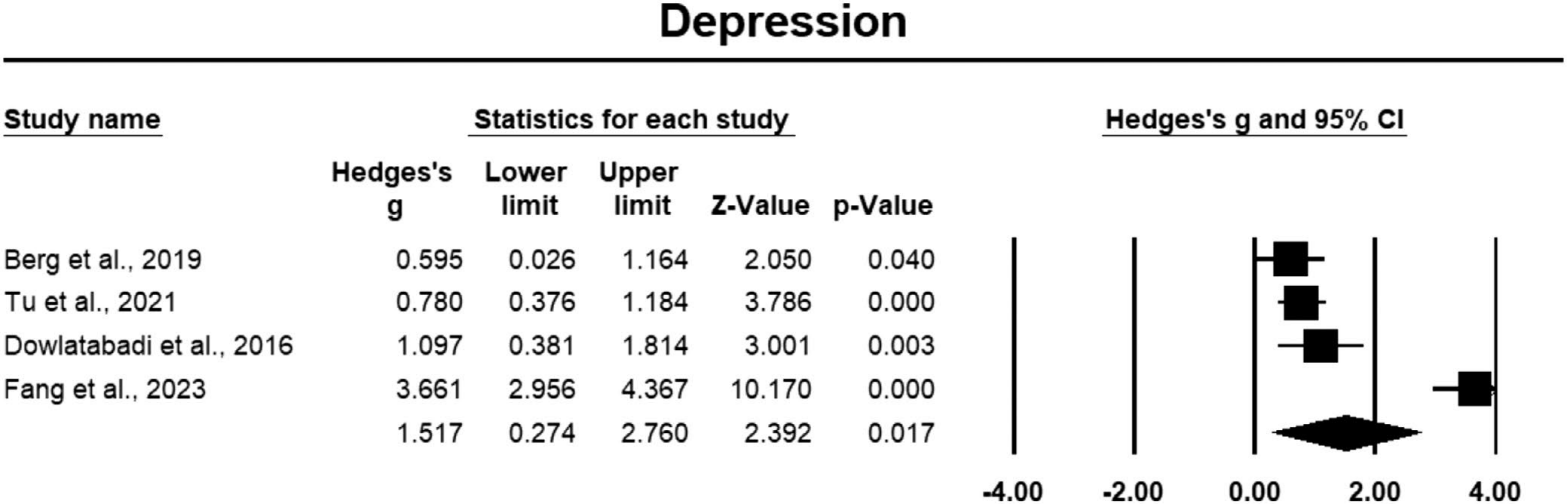
Forest plot: Effect of PPIs on depression. Study weights are reflected in the size of the squares.

Depression outcomes were measured in four studies involving a total of 269 participants with cancer [33, 38, 40, 48]. The meta‐analysis revealed a large effect size of 1.517 (Hedges' *g*) with a 95% CI of 0.274 to 2.760, which was statistically significant (*p* < 0.05), indicating that the PPIs consistently lead to significant reductions in depressive symptoms (Figure 7). Based on the sensitivity analysis, following removal of Tu et al. [40], the *p* value increased above 0.05, suggesting that without this study, the overall result is no longer statistically significant. This indicates that this study contributed to the overall significance of the results. Additionally, the removal of Fang et al. [38] led to a decrease in the overall effect size (0.784), but the *p* value remained significant (*p* < 0.05). This suggests that while Fang et al. may have influenced the effect size, the overall results remain stable and statistically significant following its removal. Furthermore, heterogeneity was high, with an *I* ^2^ value of 94.575%, reflecting substantial variability across the studies.

##### 3.3.2.2 Psychological Distress

**FIGURE 8:**
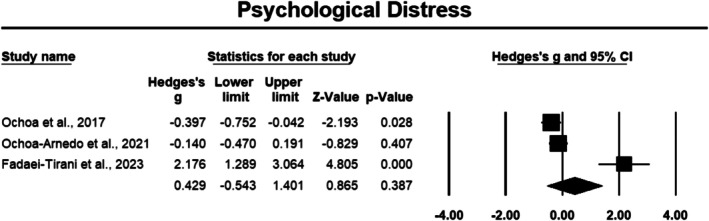
Forest plot: Effect of PPIs on psychological distress. Study weights are reflected in the size of the squares.

Psychological distress outcomes were assessed in three studies involving a total of 296 cancer participants [34, 46, 47]. The meta‐analysis produced a small effect size of 0.429 (Hedges' *g*), with a 95% CI of −0.543 to 1.401; however the result was not statistically significant (*p* > 0.05) (Figure 8). The sensitivity analysis revealed that the overall effect size for psychological distress remained stable when most studies were removed, with the exception of Fadaei‐Tirani et al. [34] which produced a significant *p* value of 0.043, suggesting that this study may have had a stronger negative influence on the overall effect size compared to the others. The outcomes exhibited high heterogeneity, with an *I* ^2^ value of 92.856%, indicating substantial variability among the studies.

##### 3.3.2.3 Post‐Traumatic Stress

Post‐traumatic stress outcomes were reported in two studies involving a total of 266 cancer survivors [46, 47]. Each study found a significant reduction in post‐traumatic stress following their respective PPIs, indicating that these interventions could alleviate post‐traumatic stress among cancer survivors.

#### 3.3.3 Physiological Health Outcomes

##### 3.3.3.1 Physical Functioning

**FIGURE 9:**
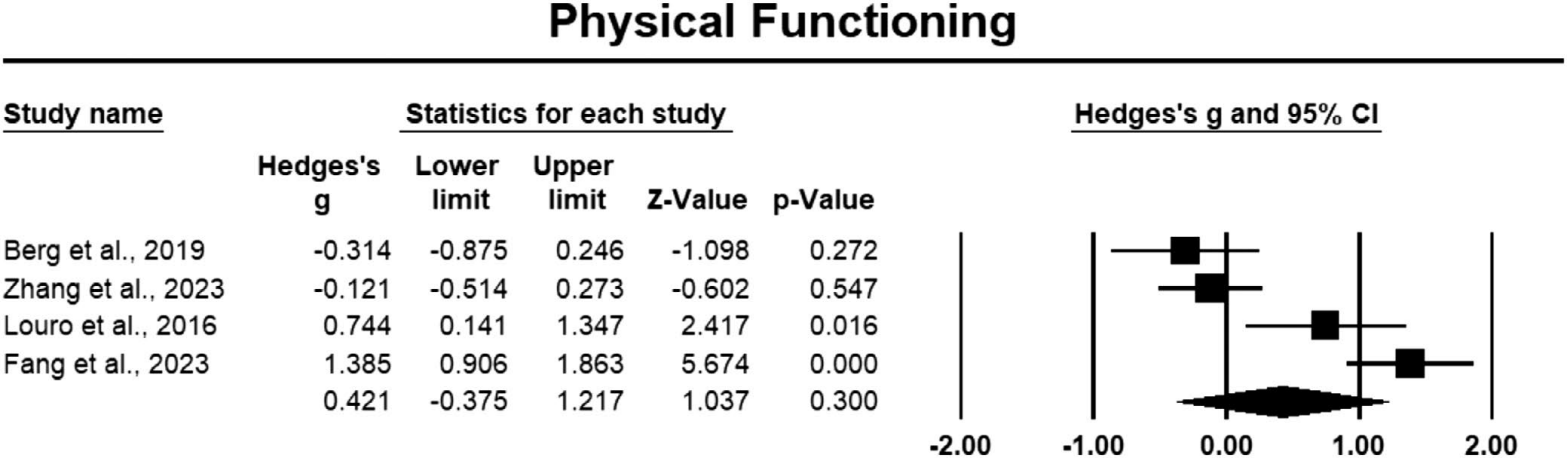
Forest plot: Effect PPIs on physical functioning. Study weights are reflected in the size of the squares.

Physical functioning following a PPI was assessed in four studies involving 278 cancer survivors [38, 41, 48, 50]. The meta‐analysis revealed a small, non‐significant effect size (Hedges' *g* = 0.421), with a 95% CI of −0.375 to 1.217 and *p* > 0.05 (Figure 9), indicating that PPIs did not consistently result in improvements in physical functioning. The sensitivity analysis revealed that removing any of these individual studies did not result in a significant change in the effect size. Heterogeneity was notably high (*I* ^2^ = 90.151%), suggesting considerable variation across the included studies.

##### 3.3.3.2 Sexual Functioning

Notably, only one study explored the impact of a PPI on sexual functioning in cancer survivors [39]. This study, which included 91 women with cervical cancer, found a significant improvement in sexual functioning.

##### 3.3.3.3 Pain

The effect of PPIs on pain outcomes was assessed in two studies involving 100 participants [48, 50]. The findings showed non‐significant improvements in pain outcomes following the interventions.

### 3.4 Results Summary

The meta‐analysis suggested that PPIs can enhance post‐traumatic growth, positive emotions, engagement, meaning, positive relationships, and depression with effect sizes ranging from moderate to large and statistically significant results (*p* < 0.05). Additionally, PPIs led to significant improvements in quality of life and psychological well‐being, highlighting their benefits for subjective well‐being. However, interventions had limited effects on accomplishment, psychological distress, and physical functioning, with outcomes not demonstrating significance. Sexual functioning improved significantly in one study, while pain and physical functioning outcomes did not show significant changes. The overall positive impact is evident, though high heterogeneity suggests variability in intervention effectiveness.

### 3.5 NHMRC FORM Synthesis

The NHMRC FORM framework was used to synthesize the results of this review (Table 5). The evidence base was rated as Good (B), with 18 studies involving 1382 cancer survivors, including a mix of Level II and Level III studies, most of which demonstrated a low risk of bias. Consistency was graded as Satisfactory (C), reflecting some variation in interventions, outcome measures, and data collection intervals across studies. Despite this heterogeneity, all studies reported statistically significant findings, reinforcing the overall reliability of the evidence. The clinical impact was deemed Good (B), with the majority of studies reporting positive outcomes and clinical significance. Interventions were generally well‐documented, and minimal dropout rates further supported the credibility of the findings. Generalisability was also rated as Good (B), with the studies being representative of the target population, covering diverse regions including the Middle East, US, UK, Europe, and Asia, and including participants at various stages of the disease.

**TABLE 5:** NHMRC FORM framework.

Component	Grade	Comments
Evidence Base	B, Good One or two level II studies with a low risk of bias or an SR/several level III studies with low risk of bias	Quantity: 18 studies, Total participants: 1382 cancer survivor participants, Level II: 9 studies, (8 low risk of bias, 1 moderate risk of bias) Level III: 9 Studies (7 low risk of bias, 2 moderate risk of bias)
Consistency	C, Satisfactory Some inconsistency reflecting genuine uncertainty around clinical question	All participants in the included studies were cancer survivors, All RCT's and quasi‐experimental studies reported statistical significance (p‐value) in findings, Wide variety of outcome measures and outcome domains, Heterogeneous interventions, Differing in frequency and duration of intervention, Varied intervals in data collection.
Clinical Impact	B, Good Substantial	Eleven studies provided justification for sample size and reported clinical significance, Majority of studies demonstrated positive change for outcome domain measured, Intervention methods were clearly described, Dropouts were minimal and predominantly attributable to death or progression of the disease, Half the studies observed follow up effects.
Generalisability	B, Good Population/s studied in the body of evidence are similar to the target population for the guideline	Population studied were representative of population of interest, Studies were conducted 7 different countries consisting of Middle East, US, UK, Europe, and Asia Majority of studies were inclusive of participants with any stage of the disease, Small to moderate sample sizes.
Grade of recommendation	B, Good Body of evidence can be trusted to guide practice in most situations	Overall, methodological quality was good, majority of studies reported positive effect, and the body of evidence was adequately representative of target population. However, interventions, outcome measures and outcome domains varied across studies.

Overall, the grade of recommendation was Good (B), indicating that the body of evidence can be trusted to guide practice in most situations. While some variation across studies was noted, the findings support the use of PPIs in cancer survivorship management.

## 4 Discussion

The objective of the systematic meta‐analysis review was to investigate the effectiveness of positive psychology interventions (PPIs) for cancer survivors. Findings highlight the potential of PPIs as a promising approach for enhancing psychological wellbeing among cancer survivors. These interventions demonstrated significant improvements in key areas such as post‐traumatic growth, positive emotions, engagement, positive relationships, meaning, and depression. While significant benefits were observed for quality of life (QOL) and sexual functioning, these outcomes were evaluated in a limited number of studies. In contrast, outcomes such as accomplishment, psychological distress, physical functioning, and pain showed limited or inconsistent effects. While the body of evidence largely indicates positive effects from PPIs, this finding is constrained by heterogeneity in intervention designs and methodologies.

Findings from this review highlight the considerable potential PPIs hold in addressing the psychological needs of cancer survivors, particularly in enhancing psychological wellbeing. Unlike traditional psycho‐oncology interventions that predominantly focus on alleviating psychological distress, PPIs aim to foster positive psychological states, such as post‐traumatic growth, positive emotions, meaning, engagement, positive relationships, accomplishment, and QOL which are critical components of flourishing while managing life with the desease [11]. Findings from the present review demonstrate that PPIs significantly improve all components of psychological wellbeing with the exception of accomplishment, which was examined in only one study. Similar to cancer, illnesses associated with significant existential distress and high symptom burden, such as HIV/AIDS, cardiac disease, and multiple sclerosis have demonstrated benefits from PPIs [51]. An RCT among HIV positive men in China reported significant improvement in depression, anxiety, and negative affect over time following a gratitude and social networking‐based PPI in comparison to the control group [52]. Correspondingly, a meta‐analysis of 22 studies involving 1222 participants with cardiovascular disease reported significant improvements in mental wellbeing and reductions in distress following PPIs, both immediately post‐intervention and at follow‐up [53]. Notably, participants with multiple sclerosis, a chronic autoimmune disease known to affect physical and mental health reported significant benefits in hope, positive affect, optimism, state and trait anxiety, general health, and resilience following a PPI [54, 55]. These findings highlight the applicability of PPIs in the adaptation and management of various life‐threatening and progressive illnesses, further reinforcing their role in promoting overall psychological wellbeing in cancer survivors.

Present meta‐analysis further suggests that the effects of PPIs on psychological distress and psychopathology symptoms are less consistent than their effects on psychological well‐being. While PPIs had a modest, non‐significant effect on psychological distress outcomes, their impact on depression was larger and statistically significant. However, sensitivity analyses revealed variability, with Fang et al. [38] study reducing the overall effect size and Tu et al. [40] study contributing to a larger observed effect size. This suggests that the effects of PPIs on depression may not be stable and are heavily influenced by individual study characteristics. These findings are consistent with broader evidence in the field, which has reported mixed results for PPIs in addressing psychological distress and psychopathology across conditions such as cancer and other chronic illnesses [12, 56]. For example, Casellas‐Grau et al. [12] observed that PPIs enhanced quality of life and resilience in cancer patients but had variable effects on distress outcomes, such as depression and anxiety. Similarly, Van Agteren et al. [56] noted that the effectiveness of PPIs in reducing psychological distress in patients with chronic illnesses was often dependent on the specific intervention components and target populations.

The variability in findings may reflect the inherent focus of positive psychology on fostering flourishing and positive emotional experience, which does not directly align with the objectives of distress‐focused interventions [9]. Additionally, the lack of standardization and clear parameters underpinning PPI design likely contributed to inconsistent outcomes [12]. This heterogeneity may in part reflect the diverse foci of the various theoretical underpinnings of different PPIs (van Agteren). Individual differences in subjective evaluations of what a person regards as key to their own functioning well or flourishing may also contribute to variability in program adherence and outcomes. For example, recent research suggests that people vary in the extent to which their happiness is determined by situational life circumstances (bottom‐up), more stable, global perceptions of one's wellbeing (top‐down) or a combination of the two [57]. At a broader level, our findings underscore the important distinction between psychological distress and wellbeing as separate, yet interrelated, constructs, each with its own spectrum and anchors [9, 11]. PPIs may be effective for the promotion and maintenance of wellbeing, whereas distress‐focused interventions may be more suitable for addressing psychopathology. This distinction highlights the importance of tailoring interventions to align with the specific psychological needs and goals of individuals.

Recent research indicates that fear of cancer recurrence (FCR) is one of the most frequently reported concerns among cancer patients and remains a significant unmet need among survivors [58, 59]. Despite evidence indicating that 59% of cancer survivors report FCR, this review did not identify any studies specifically addressing this concern [59]. The presence and severity of physical symptoms, as well as psychological distress, are strongly associated with higher levels of FCR among cancer survivors [60]. Similarly, sexual dysfunction affects a substantial proportion of cancer survivors, ranging from 30% to 100% of women with gynecological cancers, 80% of men with prostate cancer, 50%–75% of women with breast cancer, 24% of individuals with head and neck cancers, and 33% of those who survived childhood cancers [61, 62, 63]. Despite this, only one study included in this review investigated the ability of PPIs to improve sexual function [39]. Shi et al. [39] demonstrated that a self‐administered PPI effectively improved sexual functioning (post 8‐week intervention). The scarcity of research addressing both FCR and sexual dysfunction in the broader cancer population likely arises from the complex and multifaceted nature of these concerns, which encompass psychological, social, and physiological factors. Therefore, future interdisciplinary research is crucial to examine the potential of PPIs in addressing FCR and sexual dysfunction, as these issues are both prevalent and often inadequately treated.

### 4.1 Clinical Implications

The findings of this meta‐analysis highlight the potential of PPIs as a complementary approach to psycho‐oncology care, particularly in enhancing psychological wellbeing among cancer survivors. PPIs demonstrated significant benefits in areas such as post‐traumatic growth, positive emotions, meaning, engagement, and quality of life, emphasizing their role in fostering resilience and flourishing. While their efficacy in reducing psychological distress is inconsistent and addressing complex issues such as fear of cancer recurrence (FCR) and sexual dysfunction remains underexplored, PPIs may be particularly suited for the prevention and maintenance of wellbeing. To maximize their clinical utility, interventions should be tailored to individual needs, standardized in design, and integrated into multidisciplinary care models to provide a holistic approach to psychosocial care for cancer survivors.

### 4.2 Strengths and Limitations

This review is presented with several limitations. While this review adhered to best practice guidelines for the conduct and reporting of systematic reviews (PRISMA), there are limitations to consider. Despite efforts to minimize publication bias through gray literature searches and citation searching, the review included only studies published in English, potentially introducing publication and language bias in study selection, as relevant studies in languages other than English were excluded. The included studies had methodological issues, including a lack of double blinding, which can result in placebo and Hawthorne effects (while acknowledging challenges in doing so in these populations and interventions). Furthermore, several included studies reported pre–post change scores without accounting for the dependency between pre‐ and post‐intervention measures, which may have overestimated effect sizes. This analytic approach, while common, is less conservative than using follow‐up scores and should be interpreted with caution [64]. Additionally, this review did not compare the effectiveness of different types of PPIs, stratify findings by cancer stage or time since diagnosis, or by whether outcomes measured were primary or secondary. This was due to the limited number of distinct interventions and the fact that most studies did not restrict their participant populations based on cancer stage, which may have influenced the generalizability of the findings. The absence of a standardized approach across interventions further complicates the ability to draw firm conclusions. Therefore, future research should focus on identifying the optimal parameters for PPIs in cancer populations, including testing potential moderators such as delivery format, intervention length, theoretical framework, cancer type, and stage of survivorship. This would support the development of more tailored and effective evidence‐informed protocols for psychosocial care in cancer survivors.

## 5 Conclusion

In summary, PPIs largely demonstrated positive outcomes. While high heterogeneity was observed in the PPIs identified in this review, the current evidence base highlights the fundamental role of PPIs in improving wellbeing and ultimately the QOL of cancer survivors. Beyond addressing psychological wellbeing, PPIs may also provide valuable support for survivors facing challenges such as feelings of loss, uncertainty, or role transitions by fostering resilience, cultivating a sense of purpose, and promoting positive psychological states. Additionally, these interventions have the potential to address psychological distress and sexual dysfunction, warranting further research in these areas. Standardizing intervention designs, tailoring approaches to individual needs, and integrating PPIs into multidisciplinary care models will be essential to fully harness their benefits. As cancer survivorship continues to grow, PPIs represent a promising avenue to address the multifaceted psychological needs of this population and promote long‐term flourishing.

## Author Contributions

Su Ann Yeoh: conceptualization, methodology, formal analysis, data curation, project administration, writing (original draft), writing (review and editing), visualization. Alice Bowie: data curation, screening, data extraction. Tim Windsor: methodology, supervision, writing (review and editing). Hayley Russell: Supervision. Saravana Kumar: methodology, supervision, writing (review and editing). Lisa Beatty: conceptualization, methodology, supervision, writing (review and editing).

## Ethics Statement

The authors have nothing to report.

## Conflicts of Interest

The authors declare no conflicts of interest.

## Data Availability

The data that supports the findings of this study are available in within the manuscript (Appendix).

## Appendix A

**TABLE A1:** Search syntax for Ovid MEDLINE.

#	Search	Results from 07/06/2024
1	((cancer* or sarcoma* or leukemia* or tumour* or tumor* or carcinoma* or lymphoma* or myeloma* or melanoma* or adenocarcinoma* or glioma* or blastoma* or hepatoma* or mesothelioma* or teratoma*) adj2 (patient* or survivor*)).tw,kf.	505,716
2	(Positive* adj2 (psychology or psychotherap*)).tw,kf.	2388
3	Psychology, Positive/	174
4	2 or 3	2404
5	1 and 4	64

**TABLE A2:** Search syntax for Ovid Embase.

#	Search	Results from 07/06/2024
1	((cancer* or sarcoma* or leukemia* or tumour* or tumor* or carcinoma* or lymphoma* or myeloma* or melanoma* or adenocarcinoma* or glioma* or blastoma* or hepatoma* or mesothelioma* or teratoma*) adj2 (patient* or survivor*)).tw,kf.	832,661
2	(Positive* adj2 (psychology or psychotherap*)).tw,kf.	2494
3	Psychology, Positive/	723
4	2 or 3	2582
5	1 and 4	102

**TABLE A3:** Search syntax for Emcare.

#	Search	Results from 07/06/2024
1	((cancer* or sarcoma* or leukemia* or tumour* or tumor* or carcinoma* or lymphoma* or myeloma* or melanoma* or adenocarcinoma* or glioma* or blastoma* or hepatoma* or mesothelioma* or teratoma*) adj2 (patient* or survivor*)).tw,kf.	155,851
2	(Positive* adj2 (psychology or psychotherap*)).tw,kf.	2179
3	Psychology, Positive/	626
4	2 or 3	2190
5	1 and 4	50

**TABLE A4:** Search syntax for PsycINFO.

#	Search	Results from 07/06/2024
1	((cancer* or sarcoma* or leukemia* or tumour* or tumor* or carcinoma* or lymphoma* or myeloma* or melanoma* or adenocarcinoma* or glioma* or blastoma* or hepatoma* or mesothelioma* or teratoma*) adj2 (patient* or survivor*)).ti,ab.	28,002
2	(Positive* adj2 (psychology or psychotherap*)).ti,ab.	6085
3	Psychology, Positive/	6338
4	2 or 3	8438
5	1 and 4	54

**TABLE A5:** Search syntax for CINAHL.

#	Search	Results from 07/06/2024
1	“(cancer* OR sarcoma* OR leukemia* OR tumour* OR tumor* OR carcinoma* OR lymphoma* OR myeloma* OR melanoma* OR adenocarcinoma* OR glioma* OR blastoma* OR hepatoma* OR mesothelioma* OR teratoma*) n2 (patient* or survivor*)” Search mode: All text	156,247
2	(Positive n2 psycho*) Search mode: All text	46

**TABLE A6:** Search syntax for Cochran Library.

#	Search	Results from 07/06/2024
1	(cancer* OR sarcoma* OR leukemia* OR tumour* OR tumor* OR carcinoma* OR lymphoma* OR myeloma* OR melanoma* OR adenocarcinoma* OR glioma* OR blastoma* OR hepatoma* OR mesothelioma* OR teratoma*) near/2 (patient* or survivor*) Search mode: All text	757
2	(Positive near/2 psycho*) Search mode: All text	7

**TABLE A7:** Search syntax for Scopus.

#	Search	Results from 07/06/2024
1	(cancer* OR sarcoma* OR leukemia* OR tumour* OR tumor* OR carcinoma* OR lymphoma* OR myeloma* OR melanoma* OR adenocarcinoma* OR glioma* OR blastoma* OR hepatoma* OR mesothelioma* OR teratoma*) w/2 (patient* or survivor*) Search mode: Article tittle, abstract, keywords	1,005,295
2	(Positive w/2 psycho*) Search mode: Article tittle, abstract, keywords	434

*Search Syntax for Google Scholar and Google*.

Keyword include: “Cancer patient” OR “cancer survivor” AND “positive psychology”.

## Appendix B

**TABLE B1:** Data extraction table.

Study no Primary extractor Secondary extractor	Author Design Country	Sample characteristics (Age, ethnicity, duration of cancer etc.)	Intervention characteristics (Type, duration, frequency, mode, delivered by)	Positive psychology theory	Outcome measures	Primary outcomes	Secondary outcomes	Limitations

## References

[1] World Health Organisation , “New Report on Global Cancer Burden in 2022 by World Region and Human Development Level,” (2024), https://www.iarc.who.int/news‐events/new‐report‐on‐global‐cancer‐burden‐in‐2022‐by‐world‐region‐and‐human‐development‐level/.

[2] W. Linden , A. Vodermaier , R. MacKenzie , and D. Greig , “Anxiety and Depression After Cancer Diagnosis: Prevalence Rates by Cancer Type, Gender, and Age,” Journal of Affective Disorders 141, no. 2–3 (2012): 343–351. 22727334 10.1016/j.jad.2012.03.025

[3] A. Harris , J. Li , K. Atchison , et al., “Flourishing in Head and Neck Cancer Survivors,” Cancer Medicine 11, no. 13 (2022): 2561–2575. 35277936 10.1002/cam4.4636 PMC9249981

[4] S. K. Lutgendorf , A. K. Sood , and M. H. Antoni , “Host Factors and Cancer Progression: Biobehavioral Signaling Pathways and Interventions,” Journal of Clinical Oncology 28, no. 26 (2010): 4094–4099, 10.1200/JCO.2009.26.9357. 20644093 PMC2940426

[5] B. L. Andersen , C. Lacchetti , K. Ashing , et al., “Management of Anxiety and Depression in Adult Survivors of Cancer: ASCO Guideline Update,” Journal of Clinical Oncology 41, no. 18 (2023): 3426–3453. 37075262 10.1200/JCO.23.00293

[6] E. Semenenko , S. Banerjee , I. Olver , and P. Ashinze , “Review of Psychological Interventions in Patients With Cancer,” Supportive Care in Cancer 31, no. 4 (2023): 210. 36913136 10.1007/s00520-023-07675-w

[7] S. A. Yeoh , S. Webb , A. Phillips , L. S. K. Li , and S. Kumar , “Psychosocial Interventions for Ovarian Cancer Survivors: A Systematic Review,” Psycho‐Oncology 33, no. 1 (2024): e6280. 38282217 10.1002/pon.6280

[8] G. J. Westerhof and C. L. Keyes , “Mental Illness and Mental Health: The Two Continua Model Across the Lifespan,” Journal of Adult Development 17, no. 2 (2010): 110–119. 20502508 10.1007/s10804-009-9082-y PMC2866965

[9] C. L. Keyes , “The Mental Health Continuum: From Languishing to Flourishing in Life,” Journal of Health and Social Behavior 43 (2002): 207–222. 12096700

[10] J. M. Stephens , M. Iasiello , K. Ali , J. van Agteren , and D. B. Fassnacht , “The Importance of Measuring Mental Wellbeing in the Context of Psychological Distress: Using a Theoretical Framework to Test the Dual‐Continua Model of Mental Health,” Behavioral Science 13, no. 5 (2023): 436. 10.3390/bs13050436 PMC10215730 37232673

[11] M. E. Seligman , “Positive Health,” Applied Psychology 57 (2008): 3–18.

[12] A. Casellas‐Grau , A. Font , and J. Vives , “Positive Psychology Interventions in Breast Cancer. A Systematic Review,” Psycho‐Oncology 23, no. 1 (2014): 9–19. 23897834 10.1002/pon.3353

[13] K. Holtmaat , N. van der Spek , B. I. Lissenberg‐Witte , P. Cuijpers , and I. M. de Verdonck‐ Leeuw , “Positive Mental Health Among Cancer Survivors: Overlap in Psychological Well‐Being, Personal Meaning, and Posttraumatic Growth,” Supportive Care in Cancer 27 (2019): 443–450. 29959577 10.1007/s00520-018-4325-8 PMC6326009

[14] V. Lee , “The Existential Plight of Cancer: Meaning Making as a Concrete Approach to the Intangible Search for Meaning,” Supportive Care in Cancer 16 (2008): 779–785. 18197427 10.1007/s00520-007-0396-7

[15] E. Diener , S. Oishi , and R. E. Lucas , “National Accounts of Subjective Well‐Being,” American Psychologist 70, no. 3 (2015): 234–242. 25844649 10.1037/a0038899

[16] L. G. Aspinwall and R. G. Tedeschi , “The Value of Positive Psychology for Health Psychology: Progress and Pitfalls in Examining the Relation of Positive Phenomena to Health,” Annals of Behavioral Medicine 39, no. 1 (2010): 4–15. 20091429 10.1007/s12160-009-9153-0

[17] N. Park , C. Peterson , D. Szvarca , R. J. Vander Molen , E. S. Kim , and K. Collon , “Positive Psychology and Physical Health: Research and Applications,” American Journal of Lifestyle Medicine 10, no. 3 (2016): 200–206. 30202275 10.1177/1559827614550277 PMC6124958

[18] C. Cormio , F. Romito , G. Viscanti , M. Turaccio , V. Lorusso , and V. Mattioli , “Psychological Well‐Being and Posttraumatic Growth in Caregivers of Cancer Patients,” Frontiers in Psychology 5 (2014): 1342. 25477853 10.3389/fpsyg.2014.01342 PMC4238371

[19] A. Seiler and J. Jenewein , “Resilience in Cancer Patients,” Frontiers in Psychiatry 10 (2019): 208. 31024362 10.3389/fpsyt.2019.00208 PMC6460045

[20] A. K. Otto , D. Ketcher , M. Reblin , and A. L. Terrill , “Positive Psychology Approaches to Interventions for Cancer Dyads: A Scoping Review,” International Journal of Environmental Research and Public Health 19, no. 20 (2022): 13561. 36294142 10.3390/ijerph192013561 PMC9602591

[21] X. Tian , X. Zhou , M. Sun , et al., “The Effectiveness of Positive Psychological Interventions for Patients With Cancer: A Systematic Review and Meta‐Analysis,” Journal of Clinical Nursing 33 (2024): 3752–3774. 38979929 10.1111/jocn.17358

[22] L. E. Van Zyl , J. Gaffaney , L. van der Vaart , B. J. Dik , and S. I. Donaldson , “The Critiques and Criticisms of Positive Psychology: A Systematic Review,” Journal of Positive Psychology 19, no. 2 (2024): 206–235.

[23] M. J. Page , J. E. McKenzie , P. M. Bossuyt , et al., “The PRISMA 2020 Statement: An Updated Guideline for Reporting Systematic Reviews,” International Journal of Surgery 88 (2021): 105906, 10.1186/s13643-021-01626-4. 33789826

[24] A. Paez , “Gray Literature: An Important Resource in Systematic Reviews,” Journal of Evidence‐Based Medicine 10, no. 3 (2017): 233–240, 10.1111/jebm.12266. 28857505

[25] M. Law , D. Stewart , L. Letts , N. Pollock , J. Bosch , and M. Westmorland , Guidelines for Critical Review of Qualitative Studies (McMaster University Occupational Therapy Evidence‐Based Practice Research Group, 1998).

[26] N. C. Miller , S. Kumar , K. L. Pearce , and K. L. Baldock , “The Outcomes of Nature‐Based Learning for Primary School Aged Children: A Systematic Review of Quantitative Research,” Environmental Education Research 27, no. 8 (2021): 1115–1140.

[27] E. J. Tian , P. Martin , L. A. Ingram , and S. Kumar , “Effectiveness and Stakeholder Views of Community‐Based Allied Health on Acute Care Utilization: A Mixed Methods Review,” Journal of Multidisciplinary Healthcare 17 (2024): 5521–5570. 39605931 10.2147/JMDH.S489640 PMC11600924

[28] E. White , J. Zippel , and S. Kumar , “The Effect of Equine‐Assisted Therapies on Behavioural, Psychological and Physical Symptoms for Children With Attention Deficit/Hyperactivity Disorder: A Systematic Review,” Complementary Therapies in Clinical Practice 39 (2020): 101101. 32379642 10.1016/j.ctcp.2020.101101

[29] J. P. T. Higgins and S. Green , Cochrane Handbook for Systermatic Reviews of Interventions for Family (Cochrane Collaboration, 2011).

[30] J. P. Higgins , S. G. Thompson , J. J. Deeks , and D. G. Altman , “Measuring Inconsistency in Meta‐Analyses,” BMJ 327, no. 7414 (2003): 557–560. 12958120 10.1136/bmj.327.7414.557 PMC192859

[31] Australian Government. National Health Medical Research Council (NHMRC) , NHMRC Additional Levels of Evidence and Grades for Recommendations for Developers of Guidelines (NHMRC, 2009).

[32] S. Hillier , K. Grimmer‐Somers , T. Merlin , et al., “FORM: An Australian Method for Formulating and Grading Recommendations in Evidence‐Based Clinical Guidelines,” BMC Medical Research Methodology 11, no. 1 (2011): 1–8. 21356039 10.1186/1471-2288-11-23 PMC3053308

[33] M. M. Dowlatabadi , S. M. Ahmadi , M. H. Sorbi , O. Beiki , T. K. Razavi , and R. Bidaki , “The Effectiveness of Group Positive Psychotherapy on Depression and Happiness in Breast Cancer Patients: A Randomized Controlled Trial,” Electronic Physician 8, no. 3 (2016): 2175. 27123227 10.19082/2175 PMC4844485

[34] A. Fadaei‐Tirani , S. H. Alavi , H. Khosh‐Akhlagh , and Z. Ameri , “The Effectiveness of Positive Psychology Training on Distress Tolerance and Optimism in Patients With Leukemia,” International Journal of Body, Mind & Culture 10, no. 3 (2023): 2345–5802.

[35] M. Haseli , M. Afsaneh , G. Zakeripour , and S. M. Alzakerini , “Comparing the Effectiveness of Positive Education and Compassion‐Focused Therapy in Self‐Care, Self‐Worth, Well‐Being and Responsibility of Women With Breast Cancer,” Journal of Adolescent and Youth Psychological Studies (JAYPS) 4, no. 6 (2023): 66–73.

[36] R. P. Meibodi , S. D. Meftagh , and S. S. Shahangian , “The Effect of Positive Psychotherapy on Happiness and Character Strength in Cancer Patients,” Journal of Education Health Promotion 10, no. 1 (2021): 97. 34084844 10.4103/jehp.jehp_595_20 PMC8150071

[37] B. Saeedi , Z. Khoshnood , M. Dehghan , F. Abazari , and A. Saeedi , “The Effect of Positive Psychotherapy on the Meaning of Life in Patients With Cancer: A Randomized Clinical Trial,” Indian Journals 25, no. 2 (2019): 210. 10.4103/IJPC.IJPC_171_18 PMC6504746 31114105

[38] H. Fang , Y. Zeng , Y. Liu , and C. Zhu , “The Effect of the PERMA Model‐Based Positive Psychological Intervention on the Quality of Life of Patients With Breast Cancer,” Heliyon 9, no. 6 (2023): e17251. 37416631 10.1016/j.heliyon.2023.e17251 PMC10320023

[39] Y. Shi , J. Cai , Z. Wu , et al., “Effects of a Nurse‐Led Positive Psychology Intervention on Sexual Function, Depression and Subjective Well‐Being in Postoperative Patients With Early‐Stage Cervical Cancer: A Randomized Controlled Trial,” International Journal of Nursing Studies 111 (2020): 103768. 32971449 10.1016/j.ijnurstu.2020.103768

[40] M. Tu , F. Wang , S. Shen , H. Wang , and J. Feng , “Influences of Psychological Intervention on Negative Emotion, Cancer‐Related Fatigue and Level of Hope in Lung Cancer Chemotherapy Patients Based on the PERMA Framework,” Iranian Journal of Public Health 50, no. 4 (2021): 728–736. 34183922 10.18502/ijph.v50i4.5997 PMC8219625

[41] Y. Zhang , R. Tang , L. Bi , et al., “Effects of Family‐Centered Positive Psychological Intervention on Psychological Health and Quality of Life in Patients With Breast Cancer and Their Caregivers,” Supportive Care in Cancer 31, no. 10 (2023): 592. 37750931 10.1007/s00520-023-08053-2

[42] S. H. Al‐Zubaidi , A. H. Jawad , A. F. Alfarras , M. K. Obaid , and M. A. Allamy , “The Efficacy of Positive Psychology on Hope, Self‐Compassion, and Post‐Traumatic Growth in Women With Breast Cancer,” International Journal of Body, Mind & Culture 9, no. 3 (2022): 2345–5802.

[43] A. A. A. A. Alrazaq , A. A. Fadhil , N. M. Hameed , A. A. Alsaadi , S. F. Hussein , and N. A. D. Kadhum , “Comparing the Effectiveness of Positive Psychology and Gestalt Therapy on Psychological Well‐Being of Patients With Lung Cancer,” International Journal of Body, Mind & Culture 9 (2022): 2345–5802.

[44] Q. K. Kadhim , N. H. Al‐Healy , M. K. A. Al‐Maeeni , S. K. Sabri , and A. H. Adhab , “The Efficacy of Positive Group Psychotherapy on Self‐Differentiation of Patients With Prostate Cancer,” International Journal of Body, Mind & Culture 9 (2022): 2345–5802.

[45] M. V. Cerezo , M. Ortiz‐Tallo , V. Cardenal , and A. De La Torre‐Luque , “Positive Psychology Group Intervention for Breast Cancer Patients: A Randomised Trial,” Psychological Reports 115, no. 1 (2014): 44–64. 25153949 10.2466/15.20.PR0.115c17z7

[46] C. Ochoa , A. Casellas‐Grau , J. Vives , A. Font , and J.‐M. Borràs , “Positive Psychotherapy for Distressed Cancer Survivors: Posttraumatic Growth Facilitation Reduces Posttraumatic Stress,” International Journal of Clinical and Health Psychology 17, no. 1 (2017): 28–37. 30487878 10.1016/j.ijchp.2016.09.002 PMC6236322

[47] C. Ochoa‐Arnedo , A. Casellas‐Grau , M. Lleras , J. C. Medina , and J. Vives , “Stress Management or Post‐Traumatic Growth Facilitation to Diminish Distress in Cancer Survivors? A Randomized Controlled Trial,” Journal of Positive Psychology 16, no. 6 (2021): 715–725.

[48] C. J. Berg , R. C. Vanderpool , B. Getachew , et al., “A Hope‐Based Intervention to Address Disrupted Goal Pursuits and Quality of Life Among Young Adult Cancer Survivors,” Journal of Cancer Education 35 (2020): 1158–1169. 31297743 10.1007/s13187-019-01574-7 PMC6954353

[49] P. Ramachandra , S. Booth , T. Pieters , K. Vrotsou , and F. A. Huppert , “A Brief Self‐Administered Psychological Intervention to Improve Well‐Being in Patients With Cancer: Results From a Feasibility Study,” Psycho‐Oncology 18, no. 12 (2009): 1323–1326. 19180530 10.1002/pon.1516

[50] A. C. Louro , J. F. Castro , and T. B. Blasco , “Effects of a Positive Emotion‐Bases Adjuvant Psychological Therapy in Colorectal Cancer Patients: A Pilot Study,” Psicooncologia: Investigación y Clínica Biopsicosocial en Oncología 13, no. 1 (2016): 113–125.

[51] T. Barskova and R. Oesterreich , “Post‐Traumatic Growth in People Living With a Serious Medical Condition and Its Relations to Physical and Mental Health: A Systematic Review,” Disability and Rehabilitation 31, no. 21 (2009): 1709–1733. 19350430 10.1080/09638280902738441

[52] J. Li , P. Mo , C. Kahler , and J. Lau , “A Three‐Arm Randomised Controlled Trial to Evaluate the Efficacy of a Positive Psychology and Social Networking Intervention in Promoting Mental Health Among HIV‐Infected Men Who Have Sex With Men in China,” Epidemiology and Psychiatric Sciences 30 (2021): e24. 33736740 10.1017/S2045796021000081 PMC8061281

[53] K. J. Tönis , J. T. Kraiss , G. C. Linssen , and E. T. Bohlmeijer , “The Effects of Positive Psychology Interventions on Well‐Being and Distress in Patients With Cardiovascular Diseases: A Systematic Review and Meta‐Analysis,” Journal of Psychosomatic Research 170 (2023): 111328. 37098284 10.1016/j.jpsychores.2023.111328

[54] M. E. Freedman , B. C. Healy , J. C. Huffman , T. Chitnis , H. L. Weiner , and B. I. Glanz , “An At‐Home Positive Psychology Intervention for Individuals With Multiple Sclerosis: A Phase 1 Randomized Controlled Trial,” International Journal of MS Care 23, no. 3 (2021): 128–134. 34177385 10.7224/1537-2073.2020-020 PMC8218588

[55] H. Saeedi , S.‐H. M. Nasab , A. M. Zadeh , and H. A. Ebrahimi , “The Effectiveness of Positive Psychology Interventions With Islamic Approach on Quality of Life in Females With Multiple Sclerosis,” Biomedical and Pharmacology Journal 8, no. 2 (2015): 965–970.

[56] J. Van Agteren , M. Iasiello , L. Lo , et al., “A Systematic Review and Meta‐Analysis of Psychological Interventions to Improve Mental Wellbeing,” Nature Human Behaviour 5, no. 5 (2021): 631–652. 10.1038/s41562-021-01093-w 33875837

[57] E. D. Beck , F. Cheung , S. Thapa , and J. J. Jackson , “Towards a Personalized Happiness Approach to Capturing Change in Satisfaction,” Nature Human Behaviour 9 (2025): 1–14. 10.1038/s41562-025-02171-z 40316815

[58] J. Armes , M. Crowe , L. Colbourne , et al., “Patients' Supportive Care Needs Beyond the End of Cancer Treatment: A Prospective, Longitudinal Survey,” Journal of Clinical Oncology 27, no. 36 (2009): 6172–6179. 19884548 10.1200/JCO.2009.22.5151

[59] Y. L. Luigjes‐Huizer , N. M. Tauber , G. Humphris , et al., “What Is the Prevalence of Fear of Cancer Recurrence in Cancer Survivors and Patients? A Systematic Review and Individual Participant Data Meta‐Analysis,” Psycho‐Oncology 31, no. 6 (2022): 879–892. 35388525 10.1002/pon.5921 PMC9321869

[60] S. Simard , B. Thewes , G. Humphris , et al., “Fear of Cancer Recurrence in Adult Cancer Survivors: A Systematic Review of Quantitative Studies,” Journal of Cancer Survivorship 7 (2013): 300–322. 23475398 10.1007/s11764-013-0272-z

[61] R. Valpey , S. Kucherer , and J. Nguyen , “Sexual Dysfunction in Female Cancer Survivors: A Narrative Review,” General Hospital Psychiatry 60 (2019): 141–147. 31030966 10.1016/j.genhosppsych.2019.04.003

[62] B. Candy , Y. Chi , L. Graham‐Wisener , et al., “Interventions for Sexual Dysfunction Following Treatments for Cancer in Women,” Cochrane Database of Systematic Reviews 2 (2016): CD005540. 26830050 10.1002/14651858.CD005540.pub3 PMC9301918

[63] J. B. Reese , M. C. Beach , K. C. Smith , et al., “Effective Patient‐Provider Communication About Sexual Concerns in Breast Cancer: A Qualitative Study,” Supportive Care in Cancer 25 (2017): 3199–3207. 28451911 10.1007/s00520-017-3729-1 PMC5803445

[64] P. Cuijpers , E. Weitz , I. Cristea , and J. Twisk , “Pre‐Post Effect Sizes Should Be Avoided in Meta‐Analyses,” Epidemiology and Psychiatric Sciences 26, no. 4 (2017): 364–368. 27790968 10.1017/S2045796016000809 PMC6998624

